# Ensemble of Thermostatically Controlled Loads: Statistical Physics Approach

**DOI:** 10.1038/s41598-017-07462-8

**Published:** 2017-08-17

**Authors:** Michael Chertkov, Vladimir Chernyak

**Affiliations:** 1 Center for Nonlinear Studies & T-4, Theoretical Division, Los Alamos National Laboratory, Los Alamos, NM 87545 USA; 2 Skolkovo Institute of Science and Technology, 143026 Moscow, Russia; 3 Department of Chemistry, Wayne State University, 5101 Cass Ave, Detroit, MI 48202 USA

## Abstract

Thermostatically controlled loads, e.g., air conditioners and heaters, are by far the most widespread consumers of electricity. Normally the devices are calibrated to provide the so-called bang-bang control – changing from on to off, and vice versa, depending on temperature. We considered aggregation of a large group of similar devices into a statistical ensemble, where the devices operate following the same dynamics, subject to stochastic perturbations and randomized, Poisson on/off switching policy. Using theoretical and computational tools of statistical physics, we analyzed how the ensemble relaxes to a stationary distribution and established a relationship between the relaxation and the statistics of the probability flux associated with devices’ cycling in the mixed (discrete, switch on/off, and continuous temperature) phase space. This allowed us to derive the spectrum of the non-equilibrium (detailed balance broken) statistical system and uncover how switching policy affects oscillatory trends and the speed of the relaxation. Relaxation of the ensemble is of practical interest because it describes how the ensemble recovers from significant perturbations, e.g., forced temporary switching off aimed at utilizing the flexibility of the ensemble to provide “demand response” services to change consumption temporarily to balance a larger power grid. We discuss how the statistical analysis can guide further development of the emerging demand response technology.

## Introduction

Here we study thermostatically controlled loads (TCLs), or more generally loads that cycle through multiple stages, that came to the attention of the engineering community, specifically the power engineering community, in the 1980s and 1990s^1–6^. During the last decade, the subject has become a central piece of the demand response paradigm^7–11^ proposing to use power consumption flexibility of electric loads.

### TCL Models

**Figure 1:**
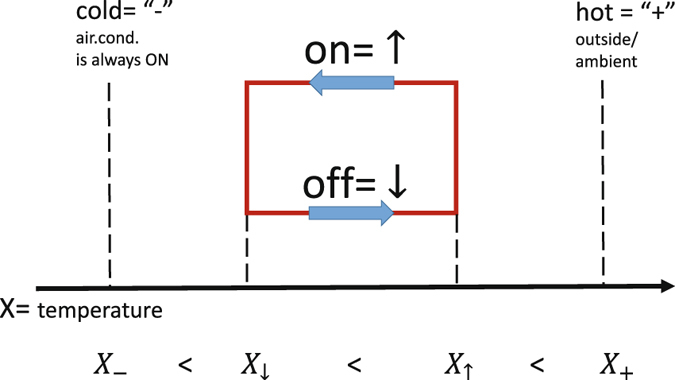
Basic TCL (cooling) model.

We look at the models from the point of view of statistical/mathematical physics. Our basic TCL model, describing the dynamics of a cooling device, e.g., an air conditioner (a heating device such as a heater or boiler can be described similarly), is stated in terms of the following stochastic system of equations, introduced and discussed in refs 1, 2, 4–6, and illustrated in Fig. (1):

1 $$\frac{dx}{dt}=-\frac{1}{\tau }\{\begin{array}{cc}x-{x}_{+}, & \sigma =\downarrow \\ x-{x}_{-}, & \sigma =\uparrow \end{array}+\xi (t),$$

2 $$\sigma (t+dt)=\{\begin{array}{cc}\downarrow , & \sigma (t)=\uparrow \,\,\&\,x < {x}_{\downarrow }\\ \uparrow , & \sigma (t)=\downarrow \,\,\&\,x > {x}_{\uparrow }\\ \sigma (t), & {\rm{otherwise}}\end{array}$$

where the dynamic variables evolving in time *t* are continuous, *x*(*t*), when describing temperature and binary, *σ*(*t*) = ↓, ↑, when describing whether the device is in the on position or the off position, respectively. The deterministic part of the model (1,2) is parameterized by the equilibrium temperatures, *x*_−_ and *x*_+_, which would be approached if the device was kept on/off, respectively, for a time longer than the relaxation time, *τ*, and the switching temperatures, *x*_↓_ and *x*_↑_, which describe when the device that was on/off at *x* > *x*_↓/↑_ switches off/on at *x* = *x*_↓/↑_. The stochastic component, imitating the effect of environment uncertainty (on temperature), is modeled by the zero mean short/white-correlated Gaussian Langevin term, *ξ*(*t*), thus fully described by the covariance, ${\mathbb{E}}[\xi ({t}_{1})\xi ({t}_{2})]=\kappa \delta ({t}_{1}-{t}_{2})$, where *κ* is the thermal drift/diffusion coefficient.

In what follows, we call the model described by Eqs (1 and 2) the “hard” model, to contrast it with the “soft” model, which assumes probabilistic, and thus soft, correction of the following type:

3 $${\rm{for}}\,\,x < {x}_{\downarrow }\,{\rm{or}}\,\,x > {x}_{\uparrow }\,{\rm{accept}}\,\sigma (t+dt)=\uparrow /\downarrow \,{\rm{from}}\,{\rm{Eq}}{\rm{.}}\,({\rm{2}})\,{\rm{with}}\,{\rm{probability}}\,\mathrm{rdt},\,{\rm{otherwise}}\,{\rm{reject}},$$

where *r* is the rate of the i.i.d. Poisson process. The soft model implements the idea of communication-limited control, where the aggregator communicates only one number—the Poisson rate *r*—to all members of the ensemble. Naturally, the hard model is recovered in the *r* → ∞ limit of the soft model. (Generalization of Eq. (3) allowing different Poisson rates depending on whether the device is switched on or off is straightforward). A model similar to our soft model was discussed in ref. 12.

## Results

We analyze the soft model, as well as its hard limit, using theoretical and computational tools of statistical physics. The main results reported in this paper are the following:

1. We derive an analytic expression for a steady probability distribution function (PDF) of the (*x*, *σ*) mixed state within the hard model and relate it to the flux of probability in this non-equilibrium setting where the detailed-balance condition is broken.
2. We analyze the Fokker–Planck (FP) equation for the PDF of the (*x*, *σ*) state in a nonsteady, transient regime, representing a solution through the spectral expansion over the (right) eigenvalues of the FP operator:
  4 $${P}_{\sigma }(x|t)=\sum _{n}\exp (-{\lambda }_{n}t){\xi }_{n}(x,\sigma ),$$
  where Re(*λ*_*n*+1_) > Re(*λ*_*n*_) > $\cdots$ > Re(*λ*_0_) = 0. Then we express *ξ*_*σ*_(*x*, *σ*) explicitly in terms of the hypergeometric functions and present *λ*_*n*_ implicitly as a solution of a system of transcendental equations (8 in the case of soft model and 4 in the case of hard model). We analyze the system of equations numerically in the case of the hard model, e.g., showing that *λ*_*n*_ for *n* ≥ 1 contains both real (decaying) and imaginary (oscillatory) parts.
3. We study the soft model in the diffusionless limit of *κ* → 0 and establish that the system mixes, i.e., a steady state is achieved, with corrections (to the steady distribution) decaying (mixing) exponentially in time, i.e., Re(*λ*_1_) > 0. We derive an explicit transcendental equation describing the spectrum. This allows for theoretical and efficient computational analysis of the spectrum, controlling relaxation. One observes that, if the switching rate is sufficiently small, *rτ* ≪ 1, oscillations of the probability distribution (as it transitions to the steady state) are suppressed, and the relaxation is split into two stages: fast but independent equilibration within the on and off states, which occurs at the rate 2/*τ*, followed by slower, non-equilibrium mixing between the on/off states, which occurs at the switching rate *r*. In contrast, if the switching rate is large, *rτ* ≫ 1, one observes persistent oscillations with the period
  5 $${t}_{dc}=\tau \,\mathrm{log}(\frac{({x}_{\uparrow }-{x}_{-})({x}_{+}-{x}_{\downarrow })}{({x}_{\downarrow }-{x}_{-})({x}_{+}-{x}_{\uparrow })}),$$
  associated with the deterministic (phase space) cycling, eventually decaying with the mixing rate estimated as ${t}_{dc}^{2}r$. The amplification factor *t*_*dc*_ *r* is interpreted as the number of the device’s cycles needed for the Poisson uncertainty to spread around the states. We also briefly discuss how the picture is affected by accounting for a small, but finite, diffusion.
4. For the soft model in the difusionless regime, we establish an explicit relationship between the dynamics of the PDF of the mixed (*x*, *σ*) state and the finite time PDF of the flux, defined as the number of cycles made in the phase space in time. The latter object measures the degree of non-equilibrium in the system (i.e., how far from detailed balance the system is). This relationship and analysis are methodologically important because, to the best of our knowledge, they are the first of this kind among other models of non-equilibrium statistical physics.

### Hard model

Stationary probability distributions for on and off states in the hard model are found analytically by solving the hard model version of the Fokker-Planck equations. (See Methods for discussion of the FP methodology and also SI for details of the stationary solution of the FP equations in the case of the hard model). Major observation, which helps to get the solution in this highly non-equilibrium case with the detailed balance broken, is the constancy of the flux in the probability space. Details are provided in SI.

#### Spectral Analysis

**Figure 2:**
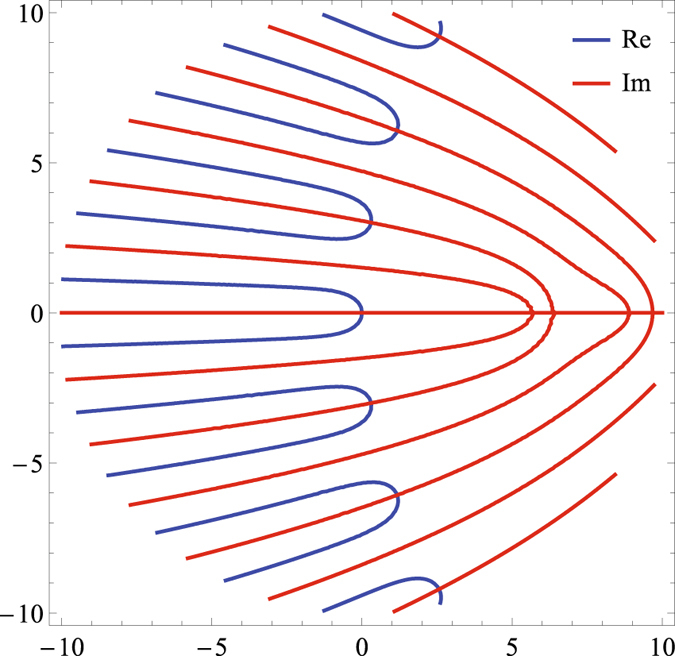
Spectral analysis of the hard model. *κ* = 0.1, *τ* = 1, *x*_−_ = −2, *x*_↓_ = −1, *x*_↑_ = 1, *x*_+_  = 2. The axes are Re[*λ*_*n*_] and Im[*λ*_*n*_]. Red and blue lines mark isolines of the real and imaginary parts of det(*M*(*λ*)), respectively (see Supplementary Information (SI) for details). Any crossing of a blue line and a red line corresponds to an eigenvalue, *λ*_*n*_.

The solution of the Fokker-Planck equations can be presented in the form of the eigenfunction expansion for the left (ket) modes of the respective FP operator^9^

6 $$(\begin{array}{c}{P}_{\uparrow }(x|t)\\ {P}_{\downarrow }(x|t)\end{array})=\sum _{n}\exp (-t{\lambda }_{n}t)(\begin{array}{c}{\xi }_{\uparrow ,n}(x)\\ {\xi }_{\downarrow ,n}(x)\end{array}),$$

where explicit expressions for left (ket) modes, *ξ*_*σ*,*n*_(*x*), (defined up to re-scaling by a convolution of the right (bra) modes with the initial condition) in terms of Kummer’s confluent hypergeometric function (further details are presented in SI). Positivity of the FP operator guarantees that the spectrum is discrete, and the eigenvalue with the lowest real part is zero. It is natural to order the eigenvalues accordingly, *λ*_0_ = 0 ≤ Re(*λ*_1_) ≤ $\cdots$ ≤ Re(*λ*_*n*_), where *n* is a non-negative integer. Notice that, contrary to what was claimed in ref. 9 and consistent with the earlier spectral analysis of ref. 6, all nonzero eigenvalues are complex, i.e., have nonzero real and imaginary parts. Eigenvalues satisfy a system of transcendental equations stated in SI explicitly as a zero condition for a determinant of a 4 × 4 complex-valued matrix. Numerical solution of the system of equations is illustrated in Fig. (2), showing isolines of real and imaginary parts of the determinant; crossing of the two identifies four (nonzero) pairs (of conjugated) eigenvalues with the smallest real parts.

### Soft Model

We have found the spectrum of the FP operator of the soft model in the diffusionless regime. Discussion of how the solution is corrected in the case of small but finite diffusion is presented in SI.

#### Fokker-Planck Spectrum

We have developed Methods described below which allowed us to derive the following closed-form transcendental equation for the spectrum of the Fokker-Planck operator in the difusionless case

7 $${(r\tau )}^{2}({\int }_{{x}_{-}}^{{x}_{\downarrow }}dx\frac{{({x}_{+}-x)}^{{\lambda }_{n}\tau }}{{(x-{x}_{-})}^{1+({\lambda }_{n}-r)\tau }})({\int }_{{x}_{\uparrow }}^{{x}_{+}}dx\frac{{(x-{x}_{-})}^{{\lambda }_{n}\tau }}{{({x}_{+}-x)}^{1+({\lambda }_{n}-r)\tau }})={(({x}_{\downarrow }-{x}_{-})({x}_{+}-{x}_{\uparrow }))}^{r\tau }\mathrm{.}$$

Derivation is done in two complimentary ways. One via direct analysis of the Fokker-planck operator itself with the properly introduced boundary conditions. The second derivation is via analysis of an individual device stochastic trajectory in the phase space. Both are detailed in the Method section.

**Figure 3:**
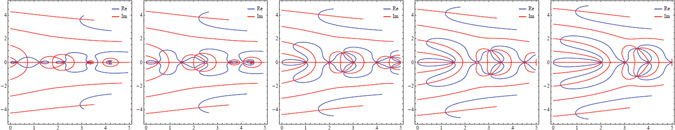
Graphical solution of Eq. (7) illustrated for *x*_−_ = −2, *x*_↓_ = −1, *x*_↑_ = 1, *x*_+_ = 2, *τ* = 1 and *r* = 0.5, 1, 1.5, 2 from left to right, respectively. Plotting conventions are the same as in Fig. 2.

Direct check shows that *λ* = 0 is a solution of Eq. (7) (as required by existence of the steady state). In general, the spectrum is complicated, as shown in Fig. (3). Of special interest is the issue of convergence of the integrals entering Eq. (7). Formally, the integrals are convergent for only Re(*λ*_*n*_) < *r*. However, the integrals allow for efficient analytic continuation beyond the condition. In fact, to get the illustrative/numerical results shown in Fig. (3), we first express the integrals in the region of their convergence via the hypergeometric functions and then use Mathematica’s ability to use known analytical properties of the hypergeometric functions to analytically continue the results beyond the constraints. In fact, it can be clearly seen that *λ*_*n*_ = *r* itself is a valid solution of Eq. (7).

We observe through numerical experiments that, as expected, only solutions with non-negative Re(*λ*_*n*_) are realized. The spectrum seen in the simulations is rich. The following features are observed. In general, solutions are complex, i.e., they contain nonzero real and imaginary parts. The real part of the eigenvalue is always positive. *λ* = 0, which corresponds to the stationary solution, is separated by a gap from the rest of the spectrum. Eigenvalues with *λ*_*n*_ ≥ *r* are always real and the *λ*_*n*_ = *r* solution is always present. When *r* is sufficiently large, an infinite sequence of solutions with Re(*λ*_*n*_) < *r* and the imaginary part increasing with *n* (by absolute value) emerges. Thus, in this regime, the long time asymptotic is an oscillatory decay controlled by *λ*_1_ with ${\rm{Im}}({\lambda }_{1}) \sim \mathrm{1/}{t}_{dc}$ and ${\rm{Re}}({\lambda }_{1}) \sim \mathrm{1/(}r{t}_{dc}^{2})$. The *rt*_*dc*_ amplification of the mixing time can be interpreted as the number of cycles needed for uncertainty associated with the device switching to spread around all the achievable states. When *r* is sufficiently small, the eigenvalue with the lowest real part is the aforementioned special one, *λ*_*n*_ = *r*, which is real, thus resulting in a purely decaying long time asymptotic.

#### Statistics of cycling time and its relation to the Fokker-Planck Spectrum

We study statistics of the flux, *ω*, defined as the number of cycles a device makes along the phase space loop (see Fig. 1) per observation time *t*, *n* = *ωt*, when the number of cycles (and the observation time) is sufficiently large. Large deviation methodology used to derive statistics of the flux is detailed in the Methods section below. Remarkably, we discover that statics of the flux is linked directly to the spectral properties of the FP operator. Establishing this type of explicit relation (see Eqs (27 and 29) below) is, to the best of our knowledge, an exception in the field of non-equilibrium statistical physics.

### Numerical Experiments

**Figure 4:**
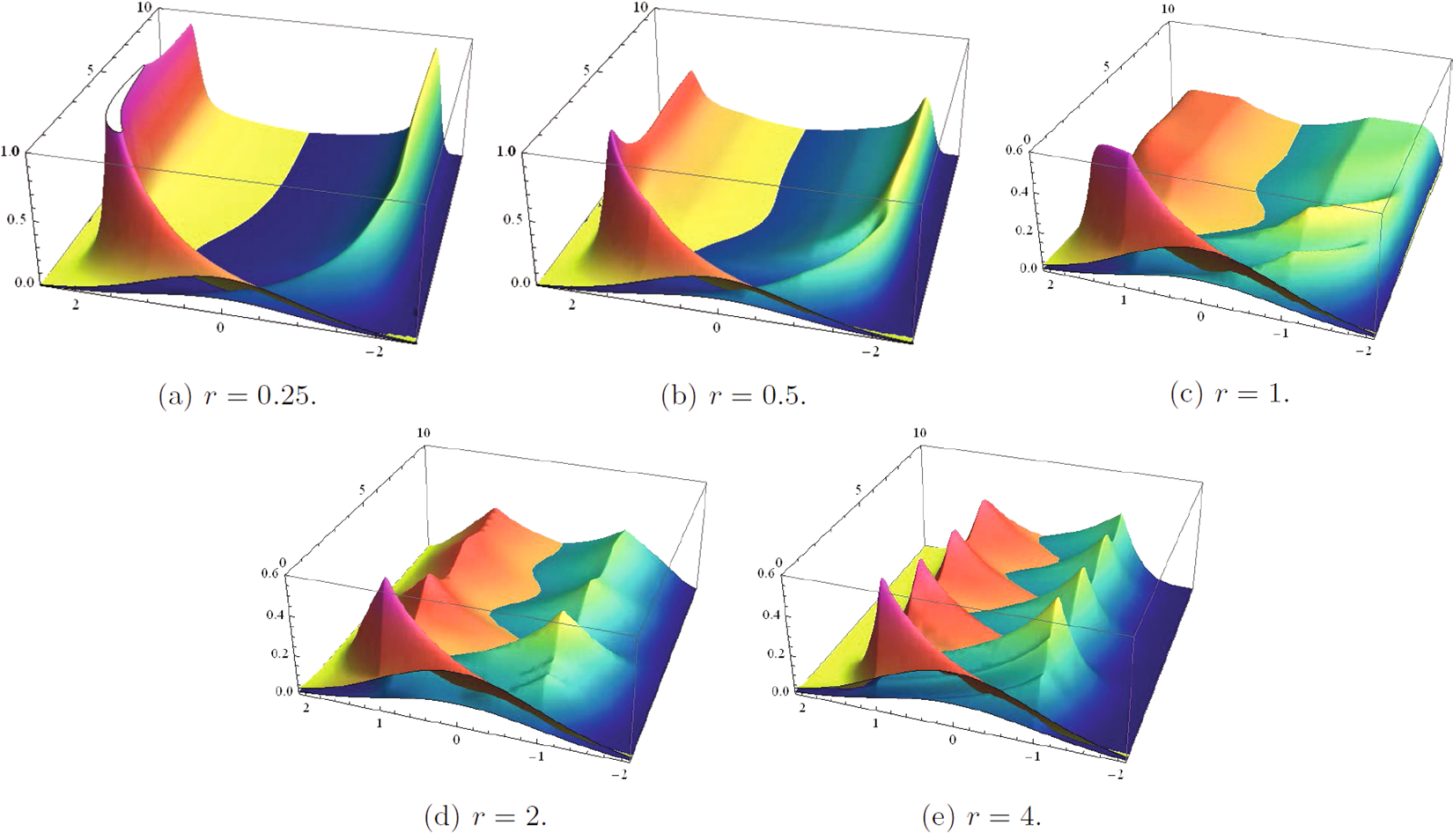
Evolution of the PDF for on/off (blue/red) states shown as a function of temperature, *x* ∈ [−2, 2], and time, *t* ∈ [0, 10]. The initial distributions are chosen to be Gaussian, with the devices split in a 0.7/0.3 proportion between the on/off states. *τ* = 1, *κ* = 0.01.

To validate and extend the results of the theoretical analysis described above, we perform direct numerical simulation of the FP Eq. (8). The results are shown in Fig. (4). We fix *τ* = 1, i.e., we measure all other temporal characteristics in units of *τ*, and choose the *x*/temperature for the experiments such that all relative temperatures are of the same order, specifically *x*_↓_ = −1, *x*_−_ = −1, *x*_+_ = 1, *x*_↑_ = 1. We also choose diffusion to be relatively small, *κ* = 0.01, in accordance with what is expected to be of interest in practical applications. Five three-dimensional subfigures of Fig. (4) show evolution of the PDFs in time starting from the initial PDFs chosen in the form of two Gaussians centered at *x* = 0 and split in a 0.7/0.3 proportion between the the on and off states The main features of the PDFs’ dynamics seen in the simulations are as follows:

- When *r* is sufficiently small, the on and off ensembles do not mix, initially equilibrating internally to the distributions picked a bit to the right/left of *x*_−_/*x*_+_ within *O*(*τ*) time. Mixing between ensembles leading to establishment of a steady distribution is seen in 0(1/*r*) time. This final relaxation to the steady state is of a pure decay type.
- Oscillations in the transients occur when *r* is increased. The first signs of the oscillations are seen at *r* < 1.
- A transition in behavior of the PDF is observed at *r* = 1. For example, for the on state, PDFs increase/decrease with *x* decrease at *x*_−_ < *x* < *x*_↓_ at *rτ* < 1/*rτ* > 1. This is seen as a gradual shift of the on state PDF center from *x*_−_ to *x*_↑_ with *r* increase.
- Oscillations mature and become quite pronounced at *r* > 1. Oscillations stop decaying with further increase of *r*, turning into a perfect oscillatory evolution with the period *t*_*dc*_ at *r* → ∞.

Notice also that all the features listed above as observed in the direct simulations are fully consistent with the theoretical and numerical results of the spectral analysis discussed above.

## Discussions

This manuscript presented detailed analysis of a model accounting for stochastic dynamics of typical thermostatic devices cycling in the mixed state, describing temperature and the switch status (on or off) of the device. Switching was modeled as a random Poisson process. We considered an ensemble of similar devices and studied the dynamics of the PDF of the mixed state (temperature and on/off status) in time. FP equations for temporal evolution of the PDF in space and time were derived and analyzed by spectral (Eulerian) and dynamic (Lagrangian) analysis. In particular, we showed that the spectral analysis is reduced to solving a transcendental equation on the eigenvalue. The equation was studied analytically in limiting cases and otherwise numerically.

A particularly interesting consequence of this analysis is establishment of the fact that Poisson transitions are sufficient for mixing the system efficiently, even in the case of zero thermal diffusivity. In this case (of the soft model) relaxation of the ensemble depends on the relation between the Poisson time (inverse switching rate), 1/*r*, and the deterministic cycling time of a device, *t*_*dc*_. If $r{t}_{dc}\gg 1$, well pronounced oscillations with the *t*_*dc*_ period decay slowly in ${t}_{dc}^{2}r$ time. Increase of the Poisson time leads to a faster mixing and the oscillations are successfully damped when *rt*_*dc*_ becomes *O*(1), however this comes on the expense of a moderate increase in the proportion of time the device spends outside of the comfort zone. We observe that this balanced regime is optimal as further increase of the Poisson time leads both to increase of the mixing time (estimated as 1/*r*) and of the proportion of time spent by the device in the discomfort zone.

In general, our analysis yielded detailed results and intuitive explanations for how the system evolves in time approaching the steady state. We also analyzed the PDF of the finite time flux, defined as the number of phase space cycles made by a device in a fixed time. We showed that the long time asymptotic of this object can be reconstructed directly from the spectrum of the FP operator. This relationship is akin to the relationship between the Eulerian (instantaneous velocity) and Lagrangian (particle dynamics) descriptions in physics, e.g., in fluid mechanics.

Results of the paper are important to both engineering (including theoretical engineering, power system engineering, and general energy systems engineering) and statistical physics communities.

The main consequence of this paper on the field of engineering is in establishing aforementioned dependencies of the mixing time of the ensemble on the parameters of the model, especially on the switching on/off rate, which is thus proposed as the major characteristic providing control of the TCL ensembles. In this context of particular interest is generalization of our results to control of more realistic ensembles, e.g., to heterogeneous/inhomogeneous ensembles including devices with different characteristics of the type discussed in ref. 13, or even more generally to coarse-grained ensembles described within the Markov decision process (MDP) framework binned/discretized in space (temperature or other exogenous characteristics) and possibly time (see e.g., refs 14, 15). Note that we discuss a related MDP approach, e.g., taking advantage of the linearly solvable control problems^14, 16, 17^, in a companion paper^18^.

Models and methods of their analysis reported in the paper are also important for the field of statistical physics because of the unusual analytical solvability of a strongly non-equilibrium problem (with detailed balance violated). Indeed, even in equilibrium statistical mechanics, where steady solution of the FP equation has a closed form Gibbs form, the spectrum of the FP operator describing PDF dynamics in general does not allow for an explicit solution, which is known for only a rather limited class of problems. Ability to find a steady-state solution extends to some special classes of non-equilibrium problems, such as queuing networks of operations research^19, 20^ and zero-range models^21, 22^ of statistical physics, where one can also analyze statistics of the measure of detailed balance violation (which can be expressed as current, entropy, or work produced)^23^. However, in these known solvable non-equilibrium statistical physics problems, as in a general equilibrium mechanics problem, finding the entire spectrum of the FP operator, or even its eigenvalue with the lowest nonzero real part, is deemed impossible. Remarkably, the soft model introduced and analyzed in this paper, in addition to being a non-equilibrium problem where steady solution and statistics of current (measuring the degree of the detailed balance violation) are known, also allows for explicit closed form expression for the spectrum. Moreover, we showed that the spectrum of the FP operator in this special mixed problem is related explicitly to statistics of the current. Extending this “complete” non-equilibrium solvability to other areas of theoretical and applied statistical mechanics, such as statistical hydrodynamics, and thus complementing the body of work on analytically tractable models of turbulence, akin to passive scalar and Burgulence theories (see e.g., ref. 24 for a review), would thus be of great interest.

## Methods

To derive results stated above we have developed two complementary methodologies: (a) spectral, Fokker-Planck analysis, and (b) device-dynamics, Lagrangian analysis.

### Fokker–Planck Equations

Here we discuss temporal dynamics of the PDF of the (*x*, *σ*) state, *P*_*σ*_(*x*|*t*), where *σ* = ↑, ↓. The so-called FP equations, governing dynamics of *P*_*σ*_(*x*|*t*), follow straightforwardly from the stochastic differential Eqs (1–3). (See classic statistical physics textbooks, e.g., refs 25–27, for a general description of the FP methodology). FP equations for the soft model (i.e. most general model considered in this manuscript) are

8 $$\frac{\partial {P}_{\uparrow }}{\partial t}=\kappa \frac{{\partial }^{2}{P}_{\uparrow }}{\partial {x}^{2}}+\frac{\partial }{\partial x}(\frac{x-{x}_{-}}{\tau }{P}_{\uparrow })-{r}_{\downarrow \uparrow }(x){P}_{\uparrow }+{r}_{\uparrow \downarrow }(x){P}_{\downarrow },$$

9 $$\frac{\partial {P}_{\downarrow }}{\partial t}=\kappa \frac{{\partial }^{2}{P}_{\downarrow }}{\partial {x}^{2}}+\frac{\partial }{\partial x}(\frac{x-{x}_{+}}{\tau }{P}_{\downarrow })-{r}_{\uparrow \downarrow }(x){P}_{\downarrow }+{r}_{\downarrow \uparrow }(x){P}_{\uparrow },$$

10 $${r}_{\uparrow \downarrow }(x)\doteq r\{\begin{array}{cc}\mathrm{1,} & x < {x}_{\downarrow }\\ \mathrm{0,} & x > {x}_{\downarrow }\end{array},\quad {r}_{\downarrow \uparrow }(x)\doteq r\{\begin{array}{cc}\mathrm{1,} & x > {x}_{\uparrow }\\ \mathrm{0,} & x < {x}_{\uparrow }\end{array}\mathrm{.}$$

The equations should be supplemented by the natural zero boundary conditions and the normalization condition, respectively:

11 $$x\to \pm \infty :\quad {P}_{\uparrow /\downarrow }(\pm \infty )=\mathrm{0,}$$

12 $${\int }_{-\infty }^{+\infty }dx({P}_{\uparrow }(t,x)+{P}_{\downarrow }(t,x))=1.$$

The general FP Eqs (8 and 9) simplify significantly in the case of the hard model. See SI for further details.

#### Spectral Derivations in the case of the diffusionless case of the soft model

If the Langevin/diffusion term is ignored, Eqs (8 and 9) combined with Eq. (6) transition to

13 $$(\begin{array}{cc}{\lambda }_{n}+{\partial }_{x}\frac{x-{x}_{-}}{\tau }-{r}_{\downarrow \uparrow }(x) & {r}_{\uparrow \downarrow }(x)\\ {r}_{\downarrow \uparrow }(x) & {\lambda }_{n}+{\partial }_{x}\frac{x-{x}_{+}}{\tau }-{r}_{\uparrow \downarrow }(x)\end{array})(\begin{array}{c}{\xi }_{\mathrm{1,}n}(x)\\ {\xi }_{\mathrm{2,}n}(x)\end{array})=0$$

Stability/spectral analysis of the diffusionless model (13), focused mainly on description of the control design for the case of the heterogeneous ensemble, was reported in ref. 13. Notice that *x* ≤ *x*_−_ and *x* ≥ *x*_+_ are not reachable in the Langevin/diffusion-free regime. In other (left, middle, and right) domains, we can write down solutions explicitly up to constants (six) to be determined as a result of imposing proper boundary conditions. Consider first the left (−) interval. Here we write

14 $${x}_{-} < x\le {x}_{\downarrow }\,:{\xi }_{1,n}(x)={c}_{1,-,n}{(x-{x}_{-})}^{-1+(r-{\lambda }_{n})\tau }$$

15 $${\xi }_{2,n}(x)={c}_{1,-,n}r\tau {({x}_{+}-x)}^{-1-{\lambda }_{n}\tau }{\int }_{{x}_{-}}^{x}dx^{\prime} \frac{{({x}_{+}-x^{\prime} )}^{{\lambda }_{n}\tau }}{{(x^{\prime} -{x}_{-})}^{1+({\lambda }_{n}-r)\tau }}$$

where we have accounted for the fact that devices cannot reach *x* = *x*_−_ in the switched-off state (one of the six boundary conditions). Respective expressions for the right interval are

16 $${x}_{\uparrow } < x\le {x}_{+}\,:{\xi }_{2,n}(x)={c}_{2,+,n}{({x}_{+}-x)}^{-1+(r-{\lambda }_{n})\tau },$$

17 $${\xi }_{1,n}(x)={c}_{2,+,n}r\tau {(x-{x}_{-})}^{-1-{\lambda }_{n}\tau }{\int }_{x}^{{x}_{+}}dx^{\prime} \frac{{(x^{\prime} -{x}_{-})}^{{\lambda }_{n}\tau }}{{({x}_{+}-x^{\prime} )}^{1+({\lambda }_{n}-r)\tau }}.$$

And solutions for the middle interval are simply

18 $${x}_{\downarrow }\le x\le {x}_{\uparrow }:{\xi }_{1,n}(x)={c}_{1,n}{(x-{x}_{-})}^{-1-{\lambda }_{n}\tau },\quad {\xi }_{2,n}(x)={c}_{2,n}{({x}_{+}-x)}^{-1-{\lambda }_{n}\tau }.$$

Relating the left, right, and middle solutions through the continuity requirement at *x*_↓_ and *x*_↑_, respectively, one arrives at Eq. (7) providing complete description of the spectrum in the diffusionless case.

### Lagrangian Representation of Device Dynamics

Consider an individual device cycling in the (*x*, *σ*) space, like a particle in physics moving in a physical space, e.g., in a fluid flow. We call this view Lagrangian to contrast it with the Eulerian view (described in the preceding Subsection of the Methods) which analyzes instantaneous probability distribution over the whole (*x*, *σ*) space. The new Lagrangian look at the dynamics brings new objects. In particular, we will analyze statistics of the number of cycles made by a device in the (*x*, *σ*) space in a finite time *t* and show that this object is directly related to the spectrum of the FP operator. Such a direct relationship between two normally unrelated (in statistical physics) objects is unusual.

Material here will be presented in three steps. First, we analyze purely deterministic Lagrangian cycling of a device in the (*x*, *σ*) space subject to the bang-bang, *r* = +∞, control. Then, we consider the case of a finite *r* where the periodicity/determinism is broken and discuss the statistics of cycling. Finally, we briefly discuss the general case. Some additional details are provided in the Supplementary Information, where we discuss statistics of the flux, relate them to the discrete spectrum of the FP operator analyzed above, and also discuss how accounting for the Langevin/stochastic perturbations modifies the picture.

#### Deterministic Cycling

*r* = +∞, *κ* = 0. In this case, the device motion is limited to the [*x*_↓_, *x*_↑_] interval. If the device was initially in the on state at *x*_↑_, its temperature decreases exponentially according to $\mathrm{log}\,\frac{{x}_{\uparrow }-{x}_{-}}{x(t)-{x}_{-}}=t/\tau$. At ${t}_{+}=\mathrm{log}\,\frac{{x}_{\uparrow }-{x}_{-}}{{x}_{\downarrow }-{x}_{-}}$, when the device temperature reaches *x*_↓_, it switches to the off state, entering the stage where temperature increases according to $\mathrm{log}\,\frac{{x}_{+}-{x}_{\uparrow }}{{x}_{+}-x(t)}=(t-{t}_{+})/\tau$. At *t*_*dc*_, defined in Eq. (5), the device reaches *x*_↑_ and then transitions to the on state, therefore completing the cycle.

Deterministic dynamics means that there is a lack of mixing within the ensemble of the devices in the *r* = ∞,*κ* = 0 case; the dynamics simply carry the initial distribution of the ensemble and return it to exactly the same initial distribution at every *t* = *nt*_*dc*_, where *n* is a positive integer.

When *κ* is small but nonzero, mixing will eventually take over. In this case, the resulting stationary PDFs in *x* space correspond to uniform distribution of the devices in time, i.e., *P*_↑/↓_(*x*) ∝ *τ*/|*x* − *x*_±_|.

#### Statistics of Cycles in diffusionless case

Stochasticity, and thus mixing, occurs in the diffusionless model by Poisson switching between on/off states. In this case, two independent Poisson-distributed intervals, and also additional traveling times when the device is outside of the [*x*_↓_, *x*_↑_] interval/level, should be added to *t*_*osc*_ to estimate the overall cycling time. Let *t* be the Poisson-distributed time for the device to transition from the on to the off state, *t*_out_ be the overall time that the device is outside of the comfort zone during the on-to-off transition, and *x* be the position where the device switches from the on state to the off state. Then the three characteristics are related to each other according to

19 $$t=\tau \,\mathrm{log}(\frac{{x}_{\downarrow }-{x}_{-}}{x-{x}_{-}}),\quad {t}_{{\rm{out}}}-t=\tau \,\mathrm{log}(\frac{{x}_{+}-x}{{x}_{+}-{x}_{\downarrow }}).$$

Excluding *x* from Eq. (19) one arrives at

20 $${t}_{{\rm{out}}}=t+\tau \,\mathrm{log}(1+\alpha (1-{e}^{-t/\tau })),\quad \alpha \doteq \frac{{x}_{\downarrow }-{x}_{-}}{{x}_{+}-{x}_{\downarrow }}.$$

Inverting relation (20), one derives

21 $$t={t}_{{\rm{out}}}+\tau \,\mathrm{log}\,\frac{1+\alpha {e}^{-{t}_{{\rm{out}}}/\tau }}{1+\alpha }.$$

Then the PDF of being outside of the comfort zone for the time *t* during the transition from on to off becomes

22 $${P}_{{\rm{out}};{\rm{down}}}({t}_{{\rm{out}}})=r\,\exp (-rt)\frac{dt}{d{t}_{{\rm{out}}}}=\frac{r}{1+\alpha {e}^{-{t}_{{\rm{out}}}/\tau }}{(\frac{1+\alpha }{{e}^{{t}_{{\rm{out}}}/\tau }+\alpha })}^{r\tau }\mathrm{.}$$

The analog of Eq. (22) for the transition from off to on is

23 $${P}_{{\rm{out}};{\rm{up}}}({t}_{{\rm{out}}})=\frac{r}{1+\beta {e}^{-{t}_{{\rm{out}}}/\tau }}{(\frac{1+\beta }{{e}^{{t}_{{\rm{out}}}/\tau }+\beta })}^{r\tau },\quad \beta \doteq \frac{{x}_{+}-{x}_{\uparrow }}{{x}_{\uparrow }-{x}_{-}}\mathrm{.}$$

Finally, the PDF of being outside of the comfort zone for the time *t* after completing *n* cycles is

24 $${P}_{{\rm{out}};{\rm{n}}}(t)={\int }_{-i\infty +\epsilon }^{+i\infty +\epsilon }\frac{ds}{2\pi i}{e}^{st}{({F}_{s}(\alpha ){F}_{s}(\beta ))}^{n},$$

25 $${F}_{s}(\alpha )\doteq {\int }_{0}^{\infty }dt{e}^{-ts}\frac{r}{1+\alpha {e}^{-t/\tau }}{(\frac{1+\alpha }{{e}^{t/\tau }+\alpha })}^{r\tau }.$$

The PDF for a device to spend time *t* making *n* cycles is *P*_out;n_(*t* − *nt*_*dc*_), where we just accounted for the fact that the total time of the device’s dynamics is the sum of the time outside of the comfort zone and the deterministic time *t*_*dc*_ of moving through the cycle within the comfort zone. Then the PDF for the device to make *n* cycles in time *t* is recomputed according to the Bias formula

26 $$P(n|t)=\frac{{P}_{{\rm{out}};{\rm{n}}}(t-n{t}_{dc})}{{\sum }_{n}{P}_{{\rm{out}};{\rm{n}}}(t-n{t}_{dc})}\mathrm{.}$$

Interested in studying the statistics of the flux, *ω*, defined as the number of cycles per observation time *t*, *n* = *ωt*, we substitute Eq. (24) in Eq. (26). Evaluating the resulting integrals in the saddle point approximation and keeping only the leading asymptotic terms, one arrives at the following asymptotic large deviation (LD) estimate for the finite time PDF of the flux:

27 $$P(\omega |t) \sim {\int }_{-i\infty +\varepsilon }^{+i\infty +\varepsilon }\frac{ds}{2\pi i}\exp (st+\omega {\rm{l}}{\rm{o}}{\rm{g}}{G}_{s}) \sim \exp (-tS(\omega )),$$

28 $${G}_{s}={(\alpha \beta )}^{\tau s}{F}_{s}(\alpha ){F}_{s}(\beta ),$$

29 $$S(\omega )=-{s}_{\ast }-\omega {G}_{{s}_{\ast }},\quad 1=-\omega {\frac{d}{ds}\mathrm{log}({G}_{s})|}_{s={s}_{\ast }}\mathrm{.}$$

Therefore, we have an implicit expression for *S*(*ω*), the so-called LD function, via a newly introduced and explicitly known *G*_*s*_ function. See SI for an extension of this analysis, showing that *G*_*s*_ is directly related to the spectrum of the FP operator.

## Electronic supplementary material

Supplementary Material
