# Supplementary material for: Ensemble of Thermostatically Controlled Loads: Statistical Physics Approach
Source: Sci Rep. 2017 Aug 17;7:8673. doi: 10.1038/s41598-017-07462-8 (PMC5561189; doi:10.1038/s41598-017-07462-8)
Supplement: Supplementary file 1 — Supplementary Material [file 41598_2017_7462_MOESM1_ESM.pdf]

Supplementary Information for
"Ensemble of Thermostatically Controlled Loads: Statistical Physics Approach"

Michael Chertkov[1,2,*], Vladimir Chernyak[3]
[1] *Center for Nonlinear Studies & T-4, Theoretical Division, Los Alamos National Laboratory, Los Alamos, NM 87545, USA*
[2] *Skolkovo Institute of Science and Technology, 143026 Moscow, Russia*
[3] *Department of Chemistry, Wayne State University, 5101 Cass Ave, Detroit, MI 48202, USA and*
[*] *Correspondence to chertkov@lanl.gov*

I. HARD MODEL

A. Fokker-Planck Equations

In the hard model limit, i.e., at $r \to \infty$, the FP Eqs. (8,9) of the main text turn into

$$\frac{\partial P_\uparrow}{\partial t} = \kappa \frac{\partial^2 P_\uparrow}{\partial x^2} + \frac{\partial}{\partial x}\left(\frac{x - x_-}{\tau} P_\uparrow\right) + J_{\uparrow\downarrow}\delta(x - x_\uparrow), \tag{1}$$

$$\frac{\partial P_\downarrow}{\partial t} = \kappa \frac{\partial^2 P_\downarrow}{\partial x^2} + \frac{\partial}{\partial x}\left(\frac{x - x_+}{\tau} P_\downarrow\right) + J_{\downarrow\uparrow}\delta(x - x_\downarrow), \tag{2}$$

supplemented by the so-called absorbing boundary conditions

$$P_\uparrow(x_\downarrow) = 0, \quad P_\downarrow(x_\uparrow) = 0, \tag{3}$$

i.e., conditions re-enforcing termination of a "particle"/air conditioner after it reaches its respective switching boundary. The "switching-induced" fluxes in Eqs. (8,9) of the main text are defined according to

$$J_{\uparrow\downarrow} = -\kappa \left(\frac{\partial P_\downarrow}{\partial x}\right)_{x = x_\uparrow} = -\kappa \partial_x P_\downarrow(x_\uparrow), \tag{4}$$
$$J_{\downarrow\uparrow} = \kappa \left(\frac{\partial P_\uparrow}{\partial x}\right)_{x = x_\downarrow} = \kappa \partial_x P_\uparrow(x_\downarrow).$$

The "instantaneous" switch terms on the right-hand sides (rhs) of Eqs. (1,2,4) reflect the absorbing nature of the jumping process, i.e., the immediate jump/switch once the hard boundary for the respective $\uparrow, \downarrow$ state is achieved, and then Eq. (4) re-enforces relationships between switching rates and respective probability fluxes at the hard boundaries. Note that the current fluxes on the rhs of Eq. (4) do not include the "advection"-induced (τ-dependent) terms, because these vanish as a result of the boundary condition (4). Notice that a system of equations for the hard model equivalent to Eqs. (1,2,3,4) was derived for the first time in [1].

B. Stationary Probability Distributions

In the stationary state of the hard model, the fluxes of probability in the $\uparrow$ and $\downarrow$ states are piecewise-constant:

$$-\kappa \frac{\partial P_{\uparrow/\downarrow}}{\partial x} + \frac{x - x_{-/+}}{\tau} P_{\uparrow/\downarrow} = \begin{cases} \mp J, & x_\downarrow \le x \le \uparrow \\ 0, & \text{otherwise} \end{cases} \tag{5}$$

where J is a positive constant, while $P_\uparrow(x)$ at $x \ge x_\uparrow$ and $P_\downarrow(x)$ at $x \le x_\downarrow$ admit, in accordance with Eq. (11) of the main text, the following equilibrium (Boltzmann) forms: $\sim \exp(-k(x - x_-)^2/(2\kappa))$. Then, solving Eq. (6) and accounting for the normalization condition, Eq. (12) of the main text, one arrives at the following explicit expressions

$$-\kappa \frac{\partial P_{\uparrow/\downarrow}}{\partial x} + \frac{x - x_{-/+}}{\tau} P_{\uparrow/\downarrow} = \begin{cases} \mp J, & x_\downarrow \le x \le \uparrow \\ 0, & \text{otherwise} \end{cases} \tag{6}$$

where J is a positive constant, while $P_\uparrow(x)$ at $x \ge x_\uparrow$ and $P_\downarrow(x)$ at $x \le x_\downarrow$ admit, in accordance with Eq. (11) of the main text, the following equilibrium (Boltzmann) forms: $\sim \exp(-k(x - x_-)^2/(2\kappa))$. Then, solving Eq. (6) and

accounting for the normalization condition, Eq. (12) of the main text, one arrives at the following explicit solution of the Fokker-Planck equations for the hard model

$$P_{\uparrow/\downarrow}(x) = \frac{J e^{-\frac{(x-x_{\mp})^2}{2\kappa\tau}}}{\kappa} g_{\uparrow/\downarrow}(x), \tag{7}$$

$$g_\uparrow(x) \doteq \begin{cases} 0, & x \le x_\downarrow \\ \int\limits_{x_\downarrow}^{x} dx' e^{-\frac{(x'-x_-)^2}{2\kappa\tau}}, & x_\downarrow \le x \le x_\uparrow \\ \int\limits_{x_\downarrow}^{x_\uparrow} dx' e^{-\frac{(x'-x_-)^2}{2\kappa\tau}}, & x_\uparrow \le x \end{cases} \tag{8}$$

$$g_\downarrow(x) = \begin{cases} 0, & x \ge x_\uparrow \\ \int\limits_{x}^{x_\uparrow} dx' e^{-\frac{(x'-x_+)^2}{2\kappa\tau}}, & x_\downarrow \le x \le x_\uparrow \\ \int\limits_{x_\downarrow}^{x_\uparrow} dx' e^{-\frac{(x'-x_-)^2}{2\kappa\tau}}, & x_\downarrow \le x \le x_\uparrow \end{cases} \tag{9}$$

$$J \doteq \frac{\kappa}{\int\limits_{-\infty}^{+\infty} dx \left(g_\uparrow(x) e^{-\frac{(x-x_-)^2}{2\kappa\tau}} + g_\downarrow(x) e^{-\frac{(x-x_+)^2}{2\kappa\tau}} \right)}. \tag{10}$$

C. Details of Spectral Computations for the Hard Model

Substituting Eq. (11) of the main text into Eqs. (24,25) of the main text, one finds that $\xi_{\uparrow/\downarrow,n}$ satisfies

$$\kappa\partial_x^2 \xi_{\uparrow,n} + \partial_x \left(\frac{x-x_-}{\tau} \xi_{\uparrow,n} \right) - \kappa(\partial_x \xi_{\downarrow,n}(x_\uparrow))\delta(x-x_\uparrow) = -\lambda_n \xi_{\uparrow,n},$$

$$\kappa\partial_x^2 \xi_{\downarrow,n} + \partial_x \left(\frac{x-x_+}{\tau} \xi_{\downarrow,n} \right) + \kappa(\partial_x \xi_{\uparrow,n}(x_\downarrow))\delta(x-x_\downarrow) = -\lambda_n \xi_{\downarrow,n}. \tag{11}$$

Strictly within the"hard" range, $x \in]x_\downarrow, x_\uparrow[$, the general solution of Eq. (11) becomes

$$\xi_{\uparrow/\downarrow,n}(x) = c_{\uparrow/\downarrow,1,n} \xi_{\uparrow/\downarrow,1,n}(x) + c_{\uparrow/\downarrow,2,n} \xi_{\uparrow/\downarrow,2,n}(x) \tag{12}$$

$$\xi_{\uparrow/\downarrow,1,n}(x) \doteq \exp\left(-\frac{x^2}{2\kappa\tau} + \frac{x x_{-/+}}{\kappa\tau} \right) {}_1F_1\left(-\frac{\lambda_n\tau}{2}, \frac{1}{2}, \frac{(x-x_{-/+})^2}{2\kappa\tau} \right) \tag{13}$$

$$\xi_{\uparrow/\downarrow,2,n}(x) \doteq \exp\left(-\frac{x^2}{2\kappa\tau} + \frac{x x_{-/+}}{\kappa\tau} \right) (x - x_{-/+})\, {}_1F_1\left(-\frac{\lambda_n\tau}{2} + \frac{1}{2}, \frac{3}{2}, \frac{(x-x_{-/+})^2}{2\kappa\tau} \right) \tag{14}$$

where c indicates yet-to-be-defined constants and ${}_1F_1(a,b,x)$ is the Kummer's confluent hypergeometric function. It is important to emphasize that in Eq. (12) we choose a pair of the homogeneous solutions that are linearly independent at all values of λ and that are not singular within the $[x_\downarrow, x_\uparrow]$ range. (Notice that even though the second terms in Eq. (12) are singular at $x = x_{-/+}$, the values are both outside of the domain of interest.) Conditions at the boundaries of the "hard" range (with the values taken at the inner sides of the range) follow from direct integrations of Eq. (12) over infinitesimally small domains around the boundary values, with the extra condition that values of all the ξ functions outside of the hard domain are zero. One arrives at

$$\xi_{\uparrow,n}(x_\downarrow) = \xi_{\downarrow,n}(x_\uparrow) = 0 \tag{15}$$

$$\kappa\partial_x \xi_{\uparrow/\downarrow,n}(x_{\uparrow/\downarrow}) + \frac{x_{\uparrow/\downarrow} - x_{-/+}}{\tau} \xi_{\uparrow/\downarrow,n}(x_{\uparrow/\downarrow}) + \kappa\partial_x \xi_{\downarrow/\uparrow,n}(x_{\uparrow/\downarrow}) = 0. \tag{16}$$

Substituting Eq. (12) into Eqs. (15,16), we arrive at four linear homogeneous relationships on four c constants:

$$M(\lambda_n) \begin{pmatrix} c_{\uparrow,1,n} \\ c_{\uparrow,2,n} \\ c_{\downarrow,1,n} \\ c_{\downarrow,2,n} \end{pmatrix} = 0, \tag{17}$$

$$M \doteq \begin{pmatrix} \xi_{\uparrow,1,n}(x_\downarrow) & \xi_{\uparrow,2,n}(x_\downarrow) & 0 & 0 \\ 0 & 0 & \xi_{\downarrow,1,n}(x_\downarrow) & \xi_{\downarrow,2,n}(x_\downarrow) \\ \kappa\partial_x \xi_{\uparrow,1,n}(x_\downarrow) + \frac{x_\downarrow - x_-}{\tau} \xi_{\uparrow,1,n}(x_\downarrow) & \kappa\partial_x \xi_{\uparrow,2,n}(x_\downarrow) + \frac{x_\downarrow - x_-}{\tau} \xi_{\uparrow,2,n}(x_\downarrow) & \kappa\partial_x \xi_{\downarrow,1,n}(x_\downarrow) & \kappa\partial_x \xi_{\downarrow,2,n}(x_\downarrow) \\ \kappa\partial_x \xi_{\uparrow,1,n}(x_\downarrow) & \kappa\partial_x \xi_{\uparrow,2,n}(x_\downarrow) & \kappa\partial_x \xi_{\downarrow,1,n}(x_\downarrow) + \frac{x_\downarrow - x_+}{\tau} \xi_{\downarrow,1,n}(x_\downarrow) & \kappa\partial_x \xi_{\downarrow,2,n}(x_\downarrow) + \frac{x_\downarrow - x_+}{\tau} \xi_{\downarrow,2,n}(x_\downarrow) \end{pmatrix} \tag{18}$$

where $M(\lambda)$ is thus an explicitly defined 4×4 matrix with coefficients that are parametrically dependent on λ. The allowed λ_n are solutions of the zero-determinant condition for the respective matrix

$$\det M(\lambda) = 0. \tag{19}$$

Even though the determinant is an explicit function of λ, resolving the zero-determinant equation explicitly does not seem feasible and we rely here on a numerical exploration of the zero-determinant Eq. (19), which is illustrated in Fig. (2) of the main text.

For the sake of completeness, notice also that the eigenvalue problem was analyzed in [2], where the author guessed that $\lambda_n = 2n/\tau$, making the first argument in the Kummer's confluent hypergeometric function a negative integer (in which case the hypergeometric functions are reduced to polynomials). However, a direct check shows that Eq. (19) is not satisfied for this guess at a finite κ.

D. Flux Generation in the Hard Model: Dynamical Viewpoint and First-Passage Time

Even though the relaxation dynamics of the hard ($r = \infty$) model and the diffusionless model are quite different, the LD function of the hard model and its relationship to the spectrum of the FP operator can be identified similarly to how they were identified for the zero diffusivity model in the main text. An appropriate dynamic object in the case of the hard model is the PDF of staying at the level σ for time t, $P_\sigma(t)$, which we can also refer to as the PDF of the first-passage time at the level σ. The PDF of making one full cycle in time t becomes $P(t) = \int_0^t dt' P_\uparrow(t') P_\downarrow(t-t')$. Respective Laplace transforms

$$G_{\sigma,s} = \int_{-\infty}^{\infty} dt e^{-st} P_\sigma(t), \quad G_s = \int_{-\infty}^{\infty} dt e^{-st} P(t) \tag{20}$$

are related to each other according to

$$G_s = G_{\downarrow,s} G_{\uparrow,s}, \tag{21}$$

which is the hard model version of Eq. (37) of the main text. The respective LD function, $S(\omega)$, is obtained from Eqs. (37,38) of the main text.

Denote by $K_\sigma(x, x_0; t)$ the solution of the FP equations (see Eqs. (24,25) of the main text) with the absorbing boundary conditions (see Eq. (26) of the main text), where the source term on the rhs of the FP equations is replaced by the initial conditions $K_\sigma(x, x_0; 0) = \delta(x - x_0)$. We then have

$$P_\downarrow(t) = -\kappa(\partial_x K_\downarrow(x, x_\downarrow; t))_{x=x_\uparrow}, \quad P_\uparrow(t) = \kappa(\partial_x K_\uparrow(x, x_\uparrow; t))_{x=x_\uparrow}. \tag{22}$$

In the regime of weak diffusion, $\kappa \ll (x_\uparrow - x_\downarrow)^2/\tau$, the first-passage time distributions can be computed explicitly using spectral decomposition and utilizing the Wentzel–Kramers–Brillouin approximation of quantum mechanics to resolve the spectrum problem.

II. SOFT MODEL

A. Relating Eulerian and Lagrangian Views

Explicit expressions discussed in the main text for the probability of advancing along the cycle in time t can also be used to compute an Eulerian object—the probability of observing a device in the state (x, σ) at a time t. This is achieved by relating the probability $P(x, \sigma | x_0, \sigma_0; t)$ of the system being in the state (x, σ) at time t, conditioned to be in state (x_0, σ_0) initially, to the probability $\hat{P}(t | x_0, \sigma_0 \to x, \sigma)$ of reaching the state (x, σ), starting at (x_0, σ_0), in time t. The former object is

$$P(x, \sigma | x_0, \sigma_0; t) = \frac{1}{\dot{x}(x, \sigma)} \hat{P}(t | x_0, \sigma_0 \to x, \sigma), \tag{23}$$

where $\dot{x}(x, \uparrow) = -(x - x_-)/\tau$ and $\dot{x}(x, \downarrow) = -(x - x_+)/\tau$ are the velocities of the deterministic motion at point x on the discrete level σ. The latter object is

$$\hat{P}(t | x_\uparrow, \uparrow \to x, \sigma) = \sum_{n=0}^{m=\lfloor t/t_{dc} \rfloor} \int_0^{t - n t_{dc}} dt' P(t' | x_\uparrow, \uparrow \to x, \sigma) P_{\text{out;n}}(t - n t_{dc} - t'), \tag{24}$$

where t_{dc}, defined in Eq. (5) of the main text, is the time needed for the device to complete one deterministic cycle within the comfort zone. $P(t'|x_0, \sigma_0 \to x, \sigma)$, entering Eq. (24), is the probability of the device transitioning from the state (x_0, σ_0) to the state (x, σ) in time t' without completing a single cycle. It is assumed in Eq. (24) that all the devices are in the $(x_\uparrow, \uparrow)$ state initially, in order to simplify the resulting expressions. Notice that at $t \to \infty$ the initial state will be forgotten, assuming sufficient mixing.

It is important to emphasize that Eq. (23) plays a crucial technical role in our approach by relating the distribution of x on the left-hand side (lhs), which is the object of our interest, to the distribution of time on the rhs, which is easy to compute using the Laplace transform technique. Such a simple relationship via just a Jacobian is due to the deterministic nature of dynamics for a given discrete level; the jumps between the discrete levels are the only source of stochasticity.

Obviously, Eq. (24) simplifies when (x, σ) is chosen to coincide with the the initial state, $(x_\uparrow, \uparrow)$. We will focus on this case, where $P(t'|x_\uparrow, \uparrow \to x_\uparrow, \uparrow) = \delta(t')$ and thus Eq. (24) becomes

$$P(x_\uparrow, \uparrow | x_\uparrow, \uparrow; t) = u^{-1} \sum_{n=0}^{m=\lfloor t/t_{dc} \rfloor} P_{\text{out;n}}(t - n t_{dc}), \tag{25}$$

with $u = |\dot{x}(x_\uparrow, \uparrow)|$.

Extending $P_{\text{out;n}}(t)$ with zero to negative times, $t < 0$, we can substitute m in Eq. (25) with ∞. Then, the Laplace transform (over time) version of Eq. (25) becomes

$$\begin{aligned} P_s(x_\uparrow, \uparrow | x_\uparrow, \uparrow) &= u^{-1} \int_0^\infty dt e^{-ts} P(x_\uparrow, \uparrow | x_\uparrow, \uparrow; t) = u^{-1} \sum_{n=0}^{\infty} (F_s(\alpha) F_s(\beta))^n \exp(-snt_{dc}) \\ &= \frac{u^{-1}}{1 - F_s(\alpha) F_s(\beta) \exp(-st_{dc})} = \frac{u^{-1}}{1 - F_s(\alpha) F_s(\beta) (\alpha\beta)^{s\tau}}, \end{aligned} \tag{26}$$

given that $F_s(\alpha) F_s(\beta) < 1$, where $F_s(\alpha)$ is defined in Eq. (34) of the Method Section of the main text. Inverse Laplace transform applied to the rhs of Eq. (26)

$$P(x_\uparrow, \uparrow | x_\uparrow, \uparrow; t) = u^{-1} \int_{-i\infty+\epsilon}^{+i\infty+\epsilon} \frac{ds}{2\pi i} e^{st} \frac{1}{1 - G_s}, \tag{27}$$

results in a sum over poles (in the domain of complex s), solving

$$1 = G_s = (\alpha\beta)^{s\tau} F_s(\alpha) F_s(\beta). \tag{28}$$

Following the same strategy and with a bit of additional effort, an explicit expression for $P(x, \sigma | x_0, \sigma_0; t)$ for a full range of arguments can be derived. Indeed, introducing the notation

$$\xi_s(x, \sigma) = \frac{1}{|\dot{x}(x, \sigma)|} \int_0^\infty dt e^{-st} P(t | x_\uparrow, \uparrow \to x, \sigma), \quad \eta_s(x_0, \sigma_0) = \int_0^\infty dt e^{-st} P(t | x_0, \sigma_0 \to x_\uparrow, \uparrow), \tag{29}$$

we arrive at the following extended version of Eq. (27):

$$P(x, \sigma | x_0, \sigma_0; t) = \int_{-i\infty+\epsilon}^{i\infty+\epsilon} \frac{ds}{2\pi i} e^{st} \eta_s(x_0, \sigma_0) \frac{1}{1 - G_s} \xi_s(x, \sigma) + \tilde{P}(x, \sigma | x_0, \sigma_0; t), \tag{30}$$

with $\tilde{P}(x, \sigma | x_0, \sigma_0; t)$ being the PDF of being in state (x, σ) at time t provided the starting state was (x_0, σ_0) and a stochastic trajectory never went through state $(x_\uparrow, \uparrow)$. A direct computation yields the following natural relationship for the eigenmodes: $\xi_{\sigma,n}(x) = \xi_{-\lambda_n}(x, \sigma)$ and $\eta_{\sigma,n}(x) = \eta_{-\lambda_n}(x, \sigma)$.

Three follow-up remarks are necessary.

First, we note that Eq. (28) is fully consistent with the spectral expression given by Eq. (18) of the main text when $s \to -\lambda_n$. To check the consistency, one just needs to change variables in the first and second integrals on the lhs of Eq. (18) of the main text according to $x = \frac{x_+ \alpha + x_- \exp(t/\tau)}{\alpha + \exp(t/\tau)}$ and $x = \frac{x_- \beta + x_+ \exp(t/\tau)}{\beta + \exp(t/\tau)}$, respectively. In fact, the dynamical derivation of Eq. (28) just presented should be considered as a rigorous proof of the spectral assumptions made in the main text.

Second, we observe that according to Eqs. (37,38) of the Methods Section of the main text, Eq. (28) and preceding remarks on the LD function, $S(\omega)$ is directly related to G_s, fully defining the spectrum of the FP operator.

Finally, the sum over poles in our description is finite. We do not prove this fact here but instead illustrate it in Appendix III A on a simpler toy model assuming an "instantaneous escape" (from the uncomfortable zone).

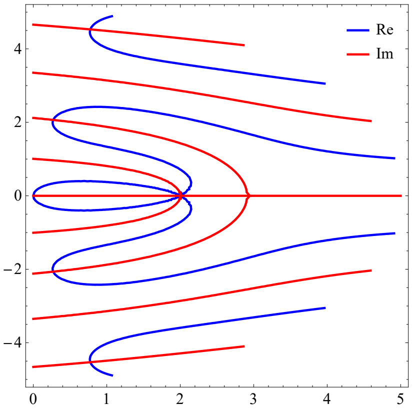

FIG. 1: Graphical solution of the spectral problem for the toy "instantaneous escape" (from the uncomfortable zone) model discussed in Appendix III A. We choose α and β parameters as described before. We also choose $\tau = 1,\ r = 2$. Plotting conventions are the same as in Fig. 2 of the main text.

III. SOFT MODEL: THE CASE OF SMALL FINITE DIFFUSIVITY

The limit $\kappa \to 0$ of small (but finite) diffusivity is important because noise/diffusion is always present in the system. The formal side of this statement is linked to the fact that the FP operator changes in transition from the diffusionless case, when it is of the first order (hyperbolic), to the small diffusion case, when it becomes of the second order (elliptic). This change in the operator order has principal consequences for the shapes of the stationary solution, the spectrum, and the dynamic behavior.

The goal of this Section is to discuss these principal changes briefly and informally, leaving more detailed mathematical analysis for future publications.

We start the discussion by mentioning that it is clear from analysis of the steady distribution conducted for the diffusionless regime above that accounting for small but finite diffusion will be important mainly at $r\tau < 1$. Indeed, in this regime, the PDFs of the on/off states are chosen in the vicinity of $x_\pm$. On the other hand, when the diffusion is strictly zero the $x_\pm$ limits are not achievable. Therefore, immediate vicinities of $x_\pm$ are controlled solely by the diffusion.

Two temporal scales are significant in this regime: first, $1/r$, which is the typical time for switching from on to off and vice versa, and second, the time for the system to stay within the on or off state. Notice that the latter time is not just τ, as one might naively suggest. Indeed, given that the majority of devices in the $r\tau < 1$ regime get rather close to $x_\pm$ while the dynamics in the vicinity of the equilibrium points slow down, the time for a device to traverse the range and then get inside the diffusion-controlled zone on the other end of the range is estimated to be $\tau\log(|x_+ - x_|/\sqrt{\kappa\tau})$. This suggests that the dynamics, spectrum, and structure of eigenvalues, discussed in the main text for the diffusionless regime, are in fact realized within the following intermediate time/transient asymptotic: $t \ll \tau\log(|x_+ - x_-|/\sqrt{\kappa\tau})$.

We now discuss the long time asymptotic regime, $t \gg \tau\log(|x_+ - x_-|/\sqrt{\kappa\tau})$, dominated by the diffusion. First, we notice that the stationary distribution, achieved as a result of a balance of the ballistic motion within a state and jumps between the states, is renormalized by small diffusion in the $|x - x_\pm| \sim \sqrt{\kappa r}$ vicinity of $x_\pm$. Similar renormalization of the x-shape by diffusion also applies to the rest of the spectrum (beyond the stationary solution). In fact, the spectrum itself is also modified in this long time regime. In particular, if r is sufficiently small, i.e., $\tau r\log(|x_+ - x_|/\sqrt{\kappa\tau}) \ll 1$, one observes a perfect equidistant spectrum $\lambda_n = n/\tau$, where $n = 0, 1, \cdots$, corresponding to separate equilibrations within the $\sigma =\uparrow$ and $\sigma =\downarrow$ ensembles.

A. Spectral Properties of a Toy "Instantaneous Escape" Model

In this Section of the Supplementary Information, we discuss the spectrum in the simple case of a toy "instantaneous escape" (from the uncomfortable zone) model, assuming that the device returns to the control zone instantaneously. See Fig. (1) for an illustration. In this case, we have

$$F_s(\alpha) = F_s(\beta) = F_s = -\frac{r}{r+s}, \tag{31}$$

and the spectral Eq. (28) simplifies to

$$(s+r)^2 = r^2 e^{-s t_{\rm dc}}. \tag{32}$$

One observes that at $r > r_{\rm cr}$ Eq. (32) has no real solutions, whereas at $r \leq r_{\rm cr}$ there is exactly one real solution, where $r_{\rm cr}$ is given implicitly by

$$(r_{\rm cr} t_{\rm dc})^2 = 4 e^{-(r_{\rm cr} t_{\rm dc}+2)}. \tag{33}$$

This implies, in particular, that $r_{\rm cr} < t_{\rm dc}^{-1}$.

Next we analyze the convergence of the spectral decomposition of the PDF of finding the device in the state (x,σ) at time t given that the device was in the state $(x_0,\downarrow)$ at the moment 0:

$$P(x,\downarrow|x_0,\downarrow;t) = \sum_n e^{-\varepsilon_n t - i\omega_n} \xi_{\downarrow,n}(x)\eta_{\downarrow,n}(x_0), \tag{34}$$

where ξ and η are the respective left and right eigenvalues of the FP operator of the toy model. Substituting s with $-\lambda_n$ in Eq. (32) and analyzing the large n part of the spectrum, one derives

$$e^{-\lambda_n t} \sim e^{\pm i\pi(2n+1)(t/t_{\rm dc})} \left(\frac{r t_{\rm dc}}{\pi(2n+1)}\right)^{2(t/t_{\rm dc})}, \quad \xi_{\downarrow,n}(x) \sim e^{\pm i\pi(\tau/t_{\rm dc})(2n+1)\alpha_\downarrow(x)} \left(\frac{(2n+1)\pi}{r t_{\rm dc}}\right)^{2\alpha_\downarrow(x)\tau/t_{\rm dc}},$$
$$\eta_{\downarrow,n}(x_0) \sim t_{\rm dc}^{-1} e^{\mp i\pi(\tau/t_{\rm dc})(2n+1)\alpha_\downarrow(x_0)} \left(\frac{(2n+1)\pi}{r t_{\rm dc}}\right)^{-2\alpha_\downarrow(x_0)\tau/t_{\rm dc}}, \quad \alpha_\downarrow(x) = \ln\left(\frac{|x_+ - x_\downarrow|}{|x_+ - x|}\right). \tag{35}$$

It follows from Eq. (35) that for given values of x and x_0 the spectral decomposition of $P(x,\downarrow|x_0,\downarrow;t)$ converges at sufficiently large t. However, if the initial distribution $P_{\downarrow,0}(x)$ is represented by a smooth function with compact support, the spectral decomposition converges at all times.

[1] Malhame, R. & Chong, C.-Y. Electric load model synthesis by diffusion approximation of a high-order hybrid-state stochastic system. *IEEE Transactions on Automatic Control* **30**, 854–860 (1985).

[2] Callaway, D. S. Tapping the energy storage potential in electric loads to deliver load following and regulation, with application to wind energy. *Energy Conversion and Management* **50**, 1389 – 1400 (2009). URL http://www.sciencedirect.com/science/article/pii/S0196890408004780.
